# Correlates of English local government use of the planning system to regulate hot food takeaway outlets: a cross-sectional analysis

**DOI:** 10.1186/s12966-019-0884-4

**Published:** 2019-12-09

**Authors:** Matthew Keeble, Jean Adams, Martin White, Carolyn Summerbell, Steven Cummins, Thomas Burgoine

**Affiliations:** 1 UKCRC Centre for Diet and Activity Research (CEDAR), MRC Epidemiology Unit, University of Cambridge, School of Clinical Medicine, Box 285 Institute of Metabolic Science, Cambridge Biomedical Campus, Cambridge, CB2 0QQ England; 2 Fuse: the centre for translational research in public health, Department of Sport and Exercise Sciences, Durham University, 32 Old Elvet, Durham, DH1 3HN England; 3 Department of Public Health, Environments & Society, Faculty of Public Health & Policy, London School of Hygiene and Tropical Medicine, 15-17 Tavistock Place, London, WC1H 9SH England

**Keywords:** Takeaway food outlet, Fast food, Food environment, Urban planning, Local government, Diet, Geography, England

## Abstract

**Background:**

Greater neighbourhood takeaway food outlet access has been associated with increased takeaway food consumption and higher body weight. National planning guidelines in England suggest that urban planning could promote healthier food environments through takeaway food outlet regulation, for example by restricting the proliferation of outlets near schools. It is unknown how geographically widespread this approach is, or local characteristics associated with its use. We aimed to address these knowledge gaps.

**Methods:**

We used data from a complete review of planning policy documents adopted by local government areas in England (*n* = 325), which contained policies for the purpose of takeaway food outlet regulation. This review classified local government area planning policies as having a health (diet or obesity) or non-health focus. We explored geographical clustering of similar planning policies using spatial statistics. We used multinomial logistic regression models to investigate whether the odds of planning policy adoption varied according to local characteristics, for example the proportion of children with excess weight or the current number of takeaway food outlets.

**Results:**

We observed clusters of local government areas with similar adopted planning policies in the North East, North West, and Greater London regions of England. In unadjusted logistic regression models, compared to local government areas with the lowest, those with highest proportion of 10–11 year olds with excess weight (OR: 25.31; 95% CI: 6.74, 94.96), and takeaway food outlet number (OR: 54.00; 95% CI: 6.17, 472.41), were more likely to have a health-focused planning policy, than none. In models adjusted for deprivation, relationships for excess weight metrics were attenuated. Compared to local government areas with the lowest, those with the highest takeaway food outlet number remained more likely to have a health-focused planning policy, than none (OR: 16.98; 95% CI: 1.44, 199.04). When local government areas were under Labour political control, predominantly urban, and when they had more geographically proximal and statistically similar areas in the same planning policy status category, they were also more likely to have health-focused planning policies.

**Conclusions:**

Planning policies for the purpose of takeaway food outlet regulation with a health focus were more likely in areas with greater numbers of takeaway food outlets and higher proportions of children with excess weight. Other characteristics including Labour political control, greater deprivation and urbanisation, were associated with planning policy adoption, as were the actions of similar and nearby local government areas. Further research should engage with local policymakers to explore the drivers underpinning use of this approach.

## Background

Health outcomes associated with excess bodyweight include type 2 diabetes, cardiovascular disease and several cancers [1, 2]. In England, around 60% of adults and 34% of 10–11 year olds are overweight or obese [3, 4]. The determinants of obesity are complex with factors at individual, community, national and transnational levels [1]. Physical access to takeaway food outlets is receiving increased public health interest. In part, this may be due to the high energy density and large portion sizes of foods typically sold, compared with meals prepared at home [5, 6]. It may also reflect higher levels of takeaway food consumption, excess body weight and greater odds of obesity in communities that have higher exposure to takeaway food outlets, even after adjusting for deprivation [7, 8].

Takeaway food outlets typically sell hot food, ordered and paid for at the counter, intended to be consumed off the premises due to limited seating provision [9, 10]. The amended “Use Class Order” used within urban planning in England, categorises these types of outlets as Class A5 "Hot food takeaways" [11]. Under this definition, there were more than 58,000 takeaway food outlets in England in 2017, rising 10% from 2014 [12], with similar estimated proliferation elsewhere such as Australia [13] and New Zealand [14]. While there have been attempts to make food provided in takeaway food outlets healthier [15], these interventions are acknowledged to be challenging [16]. An alternative approach to addressing takeaway food consumption is to use urban planning (referred to as ‘planning’ throughout) to regulate proliferation of takeaway food outlets. When permission is requested to establish a new takeaway food outlet or change the use of an existing retail unit to a takeaway food outlet, planning departments can intervene in a number of ways. There has been international precedent for this approach in the United States [17] and Ireland [18, 19]. The National Planning Policy Framework in England also advocates use of planning to maximise access to healthy food [20], however, adoption of this approach is not mandatory. Until recently it was unclear if, and how, planning was being used in this way.

In 2018, there were 325 ‘lower tier’ local government areas (formally known as local authorities) in England. These have responsibility for, amongst other things, education, waste management and planning [21, 22]. In 2018, these local government areas had an average size of 400km^2^, with a mean population of 210,492 [23]. County Councils and the City of London (*n* = 28) are not directly responsible for planning. A recent national census of adopted planning policy documents from lower tier local government areas [24], showed 164 (50.5%) had a planning policy specifically for takeaway food outlet regulation. Of these, 56 (34.1%) explicitly addressed unhealthy diets and/or obesity. In contrast, non-health focused planning policies addressed outlet design, ventilation and impact on local surroundings (e.g. litter, noise). Although this work described the nature and extent of such planning policies, it did not determine how geographically widespread their uptake had been, or local characteristics correlated with adoption.

Concerns regarding takeaway food outlet exposure and childhood obesity were noted in previous work [24], which found health-focused planning policies most often targeted this demographic. Other local characteristics, including the proportion of adults with excess weight, local political party in control, actions in neighbouring and similar local government areas, existing levels of takeaway outlets, and relative deprivation, could also influence adoption [25–27]. A greater understanding of how national planning policy guidance is adopted locally, and why some areas currently follow national directives whilst other do not, could inform future policymaking, and more effective regulation of the proliferation of takeaway food outlets through planning.

The purpose of this study was to establish if relationships exist between local government area characteristics and the adoption of planning policies to regulate takeaway food outlets.

## Methods

### Study design

Cross-sectional, geospatial analysis of local government area planning policy status, and analysis of relationships between local characteristics and planning policy status.

### Outcome variable: local government area planning policy status

The outcome of interest was local government area planning policy status, already determined through a census of planning policy documents [24].

#### Planning policy document identification

As previously described [24], we reviewed ‘Planning’ and ‘Planning Policy’ website sections of local government areas in England with planning power (*n* = 325). Local government areas publish planning policy documents on their web pages for public review. We identified relevant documents and used a key-word search strategy to identify planning policies specifically for takeaway food outlet regulation. Search terms were derived from academic literature and national guidance used by planning professionals [28, 29]. These terms were takeaway food outlet Use Class Order identifiers ‘A3’ (pre-2005) and ‘A5’ (2005 onwards) [11], ‘hot food takeaway’, ‘fast food’, ‘health’, ‘diet’ and ‘obesity’. If we were unable to identify a planning policy document on a local government area web page, we contacted planning departments directly by telephone or email to ask for assistance. Using this approach, we found relevant documents in all cases.

#### Planning policy review and categorisation

After planning policy identification and review, we categorised planning policies relevant to takeaway food outlets as ‘Non-specific’ or ‘Specific’. Non-specific policies had no explicit focus on takeaway food outlets but addressed wider food retail. For example, they provided specifications for waste management that would apply to all new food retail outlets. Specific policies referred explicitly to takeaway food outlets in their title, supporting text or planning criteria. We further sub-divided Specific planning policies into those with and without a focus on diet, obesity or diet-related disease as ‘Specific Health’ or ‘Specific Non-health’, respectively. ‘Specific Health’ planning policies commonly aimed to prevent new takeaway food outlets opening near schools, whilst ‘Specific Non-health’ planning policies described regulations related to shopfront design and traffic regulations, for example. Full details regarding the variety of takeaway food outlet focused planning policies adopted in England to date have been published previously [24].

When a relevant planning policy was not identified, local government areas were categorised as having ‘No Policy’. Due to our focus on Specific policies in this study, we combined those that had No policy (*n* = 17) with those that had a Non-specific policy (*n* = 144). Therefore, there were three outcome variable categories; ‘No, or Non-specific’, ‘Specific Non-health’ and ‘Specific Health’.

#### Planning policy year of adoption

Regardless of planning policy status, we recorded the year that each planning policy document was adopted or last revised. For those that did not have a planning policy for takeaway food outlet regulation, we used the year of their most recently adopted or last revised planning policy strategy document, which may have included planning policies focused on other food outlets; this was most commonly their Local Plan.

### Exposure variables: local government area characteristics

Based on previous research [7, 30–32], we created 11 metrics of local government area characteristics, which could plausibly influence the adoption of a takeaway food outlet focused planning policy, as follows.

#### Takeaway food outlet metrics

Data on takeaway food outlet locations were sourced from Ordnance Survey (OS) Points of Interest (POI) data for June 2014, June 2015, June 2016 and March 2017 [33]. This dataset contains information from over 170 suppliers, and is one of the most complete sources of food outlet location data available in England [34]. We extracted data on the locations of takeaway food outlets (OS POI classes ‘fast food and takeaway outlets’, ‘fast food delivery services’, ‘fish and chip shops’, ‘bakeries’), and other food outlets (cafes, convenience stores, restaurants, supermarkets and specialty outlets).

We mapped the locations of food outlets using supplied coordinates, which have a stated accuracy of 1 m [34]. We calculated three metrics per local government area: the total number of takeaway food outlets; the number per 1000 population, using data from the 2011 UK census [35]; and takeaway food outlets as a proportion (%) of all food outlets.

#### Adult and children with excess weight

We calculated the proportion (%) of children and adults with excess weight, per local government area. For children, we used data from the National Child Measurement Programme, collected annually between 2006 and 2018 [36]. Each year, children aged 4–5 and 10–11 years are weighed and measured, with this data used to derive body mass index (BMI, kg/m^2^) percentiles for these two age groups. Current UK government guidelines state that children at or above the 85th BMI percentile have excess weight [37]. For adults, we used data from the Sport England Active People Survey, collected in 2013 [38]. Self-reported height and weight data were captured via telephone survey. Local government area population weightings were applied to provide a representative sample of English adults. Those with a BMI ≥25 were determined to have excess weight.

#### Relative deprivation

We used Index of Multiple Deprivation (IMD) data [39], which is a compound measure of relative deprivation across seven domains (income deprivation, employment deprivation, crime, health deprivation and disability, education, skills and training deprivation, barriers to housing and services and living environment deprivation). We used a population weighted IMD score, averaged across lower super output areas (small administrative boundaries with a mean residential population of 1500) within local government areas.

#### Political party majority

English local elections are used to elect local councillors who represent political party interests at local level. They take place at least every four years, but not at the same time for each local government area [40]. We used the most recent local election results preceding the year of planning policy document adoption or revision to categorise local government area political party in control [41, 42]. The three main political parties represented were ‘Labour’, ‘Conservative’ and ‘Liberal Democrat’. ‘No Overall Control’ indicates that no political party won the majority of seats in an area [43]. We combined local government areas with ‘independent’ (*n* = 3) and ‘other’ (*n* = 1) political majorities in our analyses.

#### Rural/urban status

To categorise local government areas we used 2011 rural/urban classification data [44]. There were three categories; ‘Predominantly Rural’ (≥50% of residents living in rural areas), ‘Urban with Significant Rural’ (mostly urban, but 26–49% of residents living in rural areas), and ‘Predominantly Urban’ (≥74% of residents living in urban areas) [45].

#### Local government area comparators

As used elsewhere [46], the Chartered Institute of Public Finance and Accountancy (CIPFA) [47], calculate for each local government area, the 15 other most comparable local government areas in England. Calculations are based on a range of local characteristics including, for example, total population, unemployment rates and standardised mortality ratios [48]. We used CIPFA calculations from October 2018. For each local government area, we also identified neighbouring, geographically proximal local government areas who shared some part of their boundary.

For each of the 325 included local government areas, we calculated the proportion (%) of their 15 statistical comparators, and geographic neighbours, in each planning policy status category (‘No, or Non-specific’, ‘Specific Health’, ‘Specific Non-health’). In our analyses, we examined the likelihood of a local government area being in each planning policy status category per percentage point change in the proportion of comparators in each planning policy status category.

### Geospatial analyses

To understand the spatial distribution of planning policies for takeaway food outlet regulation across England, we mapped local government areas by planning policy status. We examined planning policy status spatial autocorrelation using global and local Moran’s I analyses. Moran’s I values range from − 1 (negative spatial autocorrelation, where dissimilar areas cluster), through 0 (random distribution), to + 1 (positive spatial autocorrelation, where similar areas cluster) [49]. Global Moran’s I may not be sensitive to highly localised clustering or dispersion, which would be detected through local Moran’s I analyses, and significant at *P* < 0.05. Local Moran’s I values were mapped for interpretation and identified: ‘Specific Health clusters’ (local government areas with Specific Health planning policies clustered together), ‘Specific Health outliers’ (local government areas with Specific Health planning policies surrounded by those with other policy types), ‘Specific Non-health outliers’ (local government areas with Specific Non-health planning policies surrounded by those with other policy types), and ‘Specific Non-health clusters’ (local government areas with Specific Non-health planning policies clustered together).

### Statistical analysis

We used separate multinomial logistic regression models to examine associations between exposure variables and planning policy status (‘No, or Non-specific’, ‘Specific Non-health’, ‘Specific Health’). We modelled takeaway food outlet, excess weight, and deprivation metrics as quarters, with quarter 4 representing local government areas with the highest takeaway food outlet exposure, proportion of children and adults with excess weight, or most deprivation. Adjusted models included deprivation as a covariate.

Local government areas review and adopt planning policy documents in different years. We wanted to consider exposures that were contemporaneous with year of planning policy document adoption or revision. This was possible for all metrics except those for takeaway food outlets, and child and adult excess weight measures, where data were only available from 2014, 2006 and 2013, respectively. Where local government areas had adopted a planning policy document before the first year of available exposure data (e.g. before 2014 for models including takeaway food outlet metrics), they were not included in analytic samples. Despite these exclusions, our analytic samples of local government areas remained representative of all local government areas in England in terms of characteristics for which we had data at all time points. Analyses were conducted using Stata, Version 14 (StataCorp LP., Texas) in October 2018.

## Results

### Sample characteristics

Table 1 provides descriptive statistics for local government areas. Over half of local government areas with a Specific Health planning policy were in quarter four (most exposed) with respect to total number of takeaway food outlets and number per 1000 population, and in quarter four (highest proportion) with respect to 10-11 year old children with excess weight. Most local government areas with a Specific Health planning policy were in quarter four (most deprived) with regards to relative deprivation, were under Labour political control and were predominantly urban. Of local government areas with a Specific Health planning policy, on average 43% of their geographical neighbours had the same type of policy.

**Table 1:** English local government area descriptive statistics, stratified by planning policy status for takeaway food outlet regulation, as of October 2018

		Planning Policy Status			
	N	No, or Non-Specific	Specific Non-health	Specific Health	All
Takeaway Food Outlet Metrics, Q4 ^a^					
Number, count (252–1279)	135	8 (12.1)	9 (21.9)	16 (57.1)	33 (24.4)
Number, count/1000 population (1.14–2.68) ^b^	135	6 (9.1)	12(29.3)	15 (53.5)	33 (24.4)
Proportion (%) (28.6–37.3) ^c^	135	7 (10.6)	16 (39.0)	10 (35.7)	33 (24.4)
Excess Weight proportions, Q4 ^a^					
Children 4–5 years (%) (24.1–31.5)	259	25 (20.2)	17 (20.2)	22 (43.1)	64 (24.7)
Children 10–11 years (%) (35.2–43.9)	259	16 (12.9)	19 (22.6)	27 (52.9)	62 (23.9)
Adult (%) (68.4–76.2)	156	17 (22.9)	10 (21.7)	12 (33.3)	39 (25.0)
Relative Deprivation Score					
Quarter 4, most deprived (25.24–41.99) ^a, d^	325	15 (9.3)	32 (29.6)	34 (60.7)	81 (24.9)
Political Party Majority					
No Overall Control ^e^	325	35 (21.7)	20 (18.5)	7 (12.5)	62 (19.1)
Labour	325	21 (13.0)	34 (31.5)	37 (66.1)	92 (28.3)
Conservative	325	93 (57.8)	41 (38.0)	11 (19.6)	145 (44.6)
Liberal Democrats	325	8 (5.0)	13 (18.5)	1 (1.8)	22 (6.8)
Independent and Other	325	4 (2.5)	0 (0.0)	0 (0.0)	4 (1.2)
Rural/urban Status ^f^					
Predominantly rural	325	68 (42.2)	17 (15.7)	6 (10.7)	91 (28.0)
Urban with significant rural	325	44 (27.3)	9 (8.3)	1 (1.8)	54 (16.6)
Predominantly urban	325	49 (30.4)	82 (75.9)	49 (87.5)	180 (55.4)
Proportion of Statistical Comparators by Policy Status ^g, h^ (% (SD))					
No, or Non-Specific (%)	325	60.5 (20.7)	44.8 (24.1)	27.0 (23.1)	49.5 (25.5)
Specific Non-health (%)	325	28.5 (13.9)	35.8 (13.7)	36.9 (13.5)	32.4 (14.3)
Specific Health (%)	325	10.4 (13.5)	19.4 (19.9)	36.1 (19.5)	17.8 (19.3)
Proportion of Geographical Neighbours by Policy Status ^g, i^ (% (SD))					
No, or Non-Specific (%)	325	64.4 (28.7)	47.5 (30.4)	25.6 (23.2)	52.1 (31.7)
Specific Non-health (%)	325	25.8 (23.4)	36.8 (25.8)	31.3 (23.1)	30.4 (24.6)
Specific Health (%)	325	8.5 (17.4)	15.7 (22.9)	43.2 (27.2)	16.9 (24.5)

Data are local government area number (%) unless stated. ^a^ Quarter (Q) 4 = highest. Other quarters not shown. ^b^ Number of takeaway food outlets per 1000 local government area population. ^c^ Proportion of all food retail outlets that are takeaway food outlets. ^d^ Relative Deprivation Score = measure of local deprivation. ^e^ No Overall Control = a political party did not emerge as an outright winner during local elections. ^f^ Predominantly rural = ≥50% of the population live in rural areas. Urban with significant rural = mostly urban areas with 26 to 49% of the population living in rural areas. Predominantly urban = ≥74% of the population live in urban areas. ^g^ Data = mean % (SD). ^h^ For each local government area, CIPFA (Chartered Institute of Public Finance and Accountancy) provide 15 statistical comparators based on a range of metrics. ^i^ Geographical neighbours = local government areas that share a part of their boundary. ^g^ and ^h^ should be interpreted, for example, as; among those with a Specific Health planning policy, on average, 43.2% (SD 27.2) of geographical neighbours also had a Specific Health planning policy

### Geographic distribution and spatial clustering

**Fig. 1:**
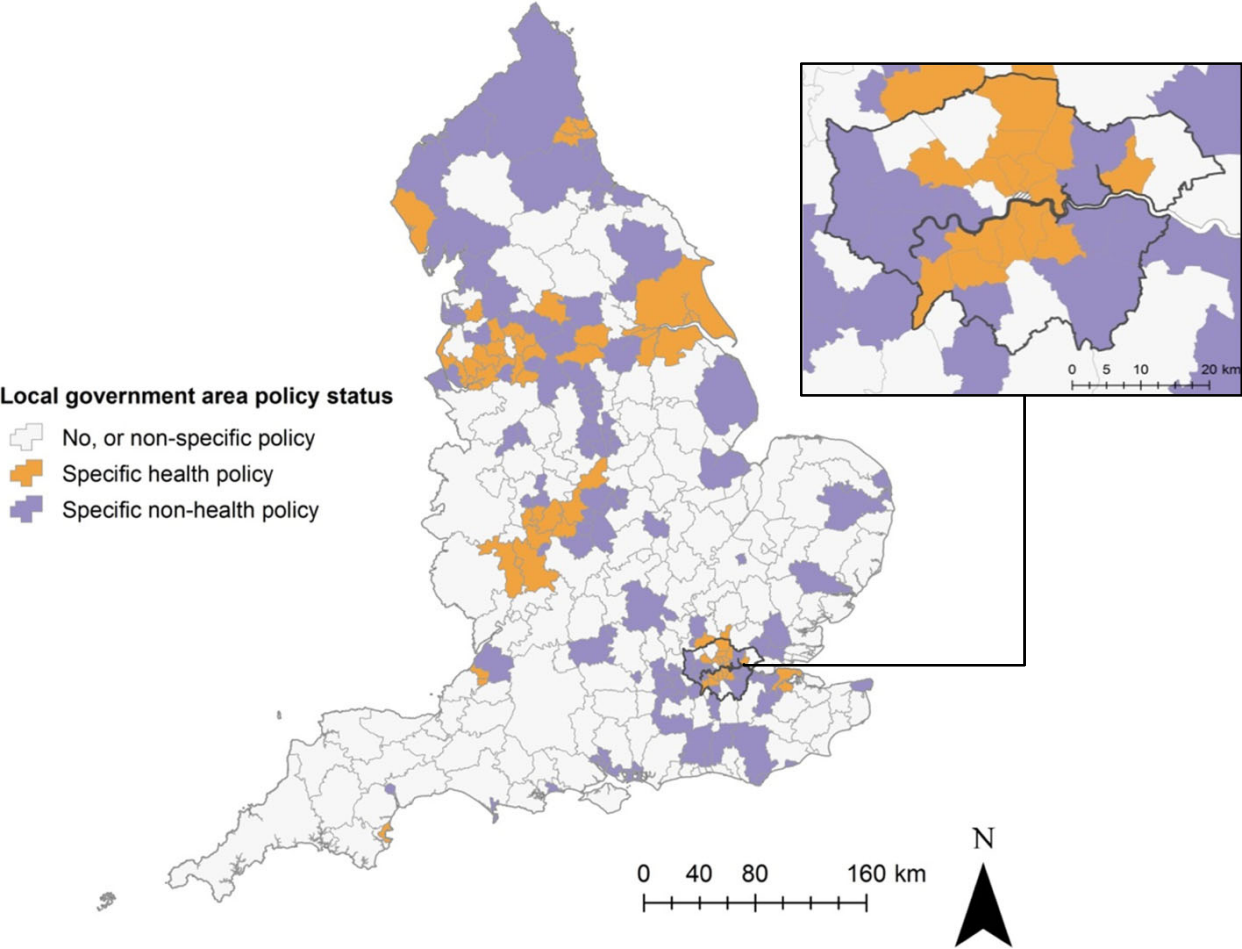
Planning policy status of local government areas in England (*n* = 325) and Greater London (inset). © Crown Copyright/database right 2019, an Ordnance Survey/EDINA supplied service

Adoption of planning policies for takeaway food outlet regulation, with and without a health focus, has been geographically widespread across England (Fig. 1). Global Moran’s I results showed significant spatial autocorrelation (*p* = 0.002), indicating a non-random spatial clustering of similar planning policies.

**Fig. 2:**
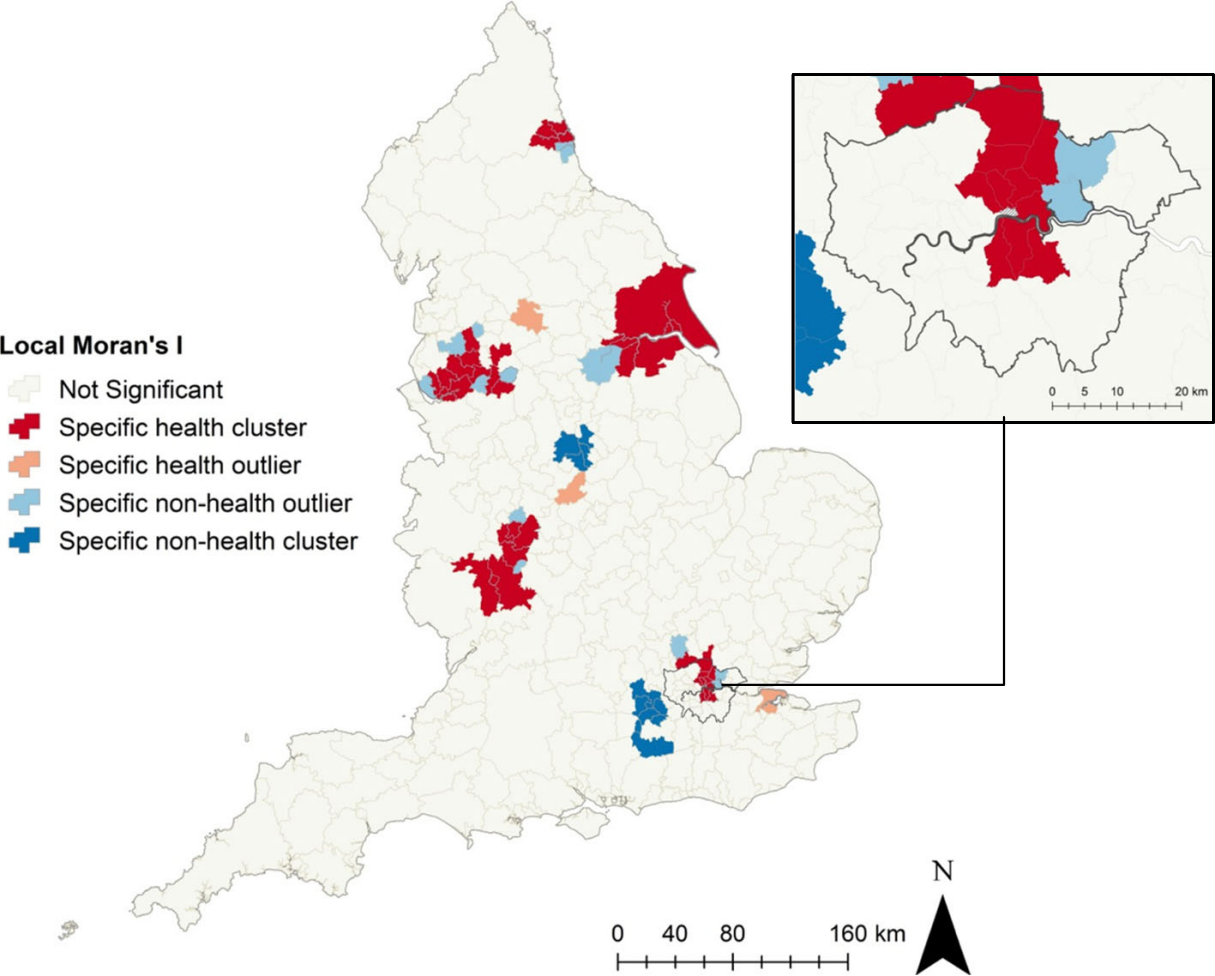
Significant, specific, health and non-health, planning policy clusters (groups of nearby local government areas with similar policy focus) and outliers (local government areas with dissimilar policy focus) across England and Greater London (inset), calculated using local Moran’s I. © Crown Copyright/database right 2019, an Ordnance Survey/EDINA supplied service

Figure 2 shows local Moran’s I results. There were significant clusters of local government areas with Specific Non-health planning policies in the Midlands, and West of Greater London. There were significant clusters of local government areas with Specific Health planning policies in the North East, North West, Yorkshire and the Humber, West Midlands and Greater London areas.

### Associations between local government characteristics and planning policy adoption status

#### Relative deprivation

Greater relative deprivation was associated with greater odds of Specific planning policy adoption (Table 2). Compared to local government areas in quarter one (least deprived), those in quarter four (most deprived) had 4.26 (95% CI: 1.97, 9.19) greater odds of Specific Non-health, and had 40.80 (95% CI: 10.99, 151.47) greater odds of Specific Health planning policy adoption.

**Table 2:** Associations of English local government area characteristics and planning policy status, estimated using unadjusted and adjusted multinomial logistic regression models (*n* = 325)

	Specific Non-health Planning Policy Status				Specific Health Planning Policy Status			
	Unadjusted		Adjusted		Unadjusted		Adjusted	
	OR	95% CI	OR	95% CI	OR	95% CI	OR	95% CI
Relative Deprivation Score ^a^								
Q1, least deprived (5.00–12.85)	ref	–	ref	–	ref	–	ref	–
Q2 (12.86–18.01)	0.80	0.40, 1.60	–	–	2.76	0.69, 11.01	–	–
Q3 (18.02–25.23)	1.40	0.71, 2.73	–	–	4.95	1.29, 18.91	–	–
Q4, most deprived (25.24–41.99)	4.26	1.97, 9.19	–	–	40.80	10.99, 151.47	–	–
Political Party Majority ^b, c^								
No Overall Control	ref	–	ref	–	ref	–	ref	–
Labour	2.83	1.30, 6.13	2.07	0.92, 4.68	8.80	3.33, 23.29	5.17	1.84, 14.49
Conservative	0.77	0.39, 1.49	0.97	0.47, 2.00	0.59	0.21, 1.64	1.23	0.40, 3.79
Liberal Democrat	2.84	1.00, 8.02	3.15	1.07, 9.31	0.62	0.67, 5.82	0.90	0.91, 9.01
Rural/urban Status ^d^								
Predominantly rural	ref	–	ref	–	ref	–	ref	–
Urban with significant rural	0.82	0.33, 1.99	0.76	0.31, 1.89	0.25	0.02, 2.21	0.25	0.02, 2.25
Predominantly urban	6.69	3.53, 12.68	5.63	2.88, 11.00	11.33	4.49, 28.54	5.51	2.05, 14.87
Statistical Comparator Policy Status ^e, f^								
No, or Non-specific	0.97	0.96, 0.98	0.97	0.96, 0.98	0.94	0.92, 0.95	0.95	0.93, 0.97
Specific Non-health	1.01	1.01, 1.05	1.02	1.00, 1.04	1.04	1.02, 1.06	1.00	0.98, 1.03
Specific Health	1.03	1.01, 1.05	1.02	1.00, 1.04	1.07	1.05, 1.09	1.05	1.03, 1.07
Geographical Neighbour Policy Status ^e, g^								
No, or Non-specific	0.98	0.97, 0.98	0.98	0.97, 0.99	0.95	0.93, 0.96	0.95	0.94, 0.97
Specific Non-health	1.01	1.00, 1.02	1.01	1.00, 1.02	1.00	0.99, 1.02	1.00	0.99, 1.02
Specific Health	1.02	1.00, 1.03	1.01	1.00, 1.03	1.05	1.04, 1.07	1.05	1.03, 1.06

Odds ratios (ORs) and 95% CIs relative to reference group (ref). ^a^Relative Deprivation Score = measure of local deprivation. ^b^For 'Political Party Majority', 'Independent and other' were not included in analysis due to low representation (*n *= 4). Analytic sample for this model, *n* = 321. ^c^No Overall Control = a political party did not emerge as an outright winner during local elections. ^d^ Predominantly rural = ≥50% of the population live in rural areas. Urban with significant rural = mostly urban areas with 26 to 49% of the population living in rural areas. Predominantly urban = ≥74% of the population live in urban areas. ^e^ORs and 95% CIs per one percentage point increase in the percentage of comparators with ‘No, or Non-specific’, ‘Specific Non-health’ or ‘Specific Health’ planning policy in place. ^f^For each local government area, CIPFA (Chartered Institute of Public Finance and Accountancy) provide 15 nearest statistical comparators based on a range of metrics, independent of geographical location. ^g^Geographical neighbours are local government areas that share a part of their boundary

#### Political party majority

In the unadjusted model, compared to local government areas with No Overall Control, those under Labour (OR: 2.83; 95% CI: 1.30, 6.13), or Liberal Democrat (OR: 2.84; 95% CI: 1.00, 8.02) control had greater odds of Specific Non-health planning policy adoption (Table 2). Those under Labour control had greater odds of Specific Health planning policy adoption (OR: 8.80; 95% CI: 3.33, 23.29).

After adjusting for deprivation, only associations with Labour control remained significant for Specific Health planning policy adoption (OR: 5.17; 95% CI: 1.84, 14.49). The effect of Liberal Democrat control on Specific Non-health planning policy adoption was strengthened (OR: 3.15; 95% CI: 1.07, 9.31).

#### Rural/urban status

In the unadjusted model, local government areas had 6.69 (95% CI: 3.53, 12.68) greater odds of Specific Non-health, and 11.33 (95% CI: 4.49, 28.54) greater odds of Specific Health planning policy adoption when they were predominantly urban compared to when they were predominantly rural (Table 2). Associations were attenuated but remained significant in the adjusted model. Predominantly urban areas had 5.63 (95% CI: 2.88, 11.00) greater odds of Specific Non-health, and 5.51 (95% CI: 2.05, 14.87) greater odds of Specific Health planning policy adoption.

#### Local government area comparators

In the unadjusted model, a greater proportion of local government area statistical comparators with a Specific Non-health planning policy was associated with increased odds of Specific Non-health (OR: 1.01 per additional percentage point; 95% CI: 1.01, 1.05), or Specific Health (OR: 1.04; 95% CI: 1.02, 1.06) planning policy adoption. A greater proportion of statistical comparators with a Specific Health planning policy was associated with increased odds of Specific Non-health (OR: 1.03 per additional percentage point; 95% CI: 1.01, 1.05), or Specific Health (OR: 1.07; 95% CI: 1.05, 1.09) planning policy adoption. Adjusted results were not materially different, and similar associations were observed for geographical neighbours (Table 2).

#### Takeaway food outlet metrics

In the unadjusted model, greater takeaway food outlet number, density per 1000 population and proportion of outlets, were associated with planning policy adoption, with the suggestion of a dose-response relationship for each (Table 3). Compared to those with the fewest, local government areas with the most takeaway food outlets had 5.06 (95% CI: 1.37, 18.57) greater odds of Specific Non-health, and 54.00 (95% CI: 6.17, 472.41) greater odds of Specific Health planning policy adoption. Those with the greatest density per 1000 population had 9.33 (95% CI: 2.49, 34.87) greater odds of Specific Non-health, and 23.33 (95% CI: 5.09, 106.81) greater odds of Specific Health planning policy adoption. Those with the greatest proportion had 19.80 (95% CI: 4.46, 87.80) greater odds of Specific Non-health, and 7.42 (95% CI: 1.90, 28.93) greater odds of Specific Health planning policy adoption.

**Table 3:** Associations of takeaway food outlet metrics and planning policy status in English local government areas, estimated using unadjusted and adjusted multinomial logistic regression models (*n* = 135)

	Specific Non-health Planning Policy Status				Specific Health Planning Policy Status			
	Unadjusted		Adjusted		Unadjusted		Adjusted	
	OR	95% CI	OR	95% CI	OR	95% CI	OR	95% CI
Number, count^a^								
Q1 (0–76)	ref	–	ref	–	Ref	–	ref	–
Q2 (77–120)	4.21	1.36, 13.07	5.10	1.46, 17.74	6.75	0.69, 65.78	5.35	0.48, 59.28
Q3 (121–251)	3.30	1.01, 10.71	2.52	0.65, 9.74	12.60	1.41, 112.39	4.85	0.44, 52.70
Q4 (252–1279)	5.06	1.37, 18.57	3.93	0.78, 19.64	54.00	6.17, 472.41	16.98	1.44, 199.04
Number, count/1000 population ^a, b^								
Q1 (0.0–0.75)	ref	–	ref	–	ref	–	ref	–
Q2 (0.76–0.96)	1.90	0.59, 6.17	2.15	0.57, 8.03	1.27	0.23, 6.93	0.57	0.08, 3.79
Q3 (0.97–1.13)	6.53	1.97, 21.65	7.77	1.85, 32.64	6.53	1.41, 30.26	2.05	0.33, 12.50
Q4 (1.14–2.68)	9.33	2.49, 34.87	10.80	1.90, 61.28	23.33	5.09, 106.81	4.44	0.63, 30.95
Proportion (%) ^a, c^								
Q1 (0.0–20.5)	ref	–	ref	–	ref	–	ref	–
Q2 (20.6–24.3)	6.11	1.50, 24.93	5.89	1.36, 25.48	1.52	0.38, 6.09	0.88	0.18, 4.27
Q3 (24.4–28.5)	5.41	1.29, 22.69	4.95	1.10, 22.22	2.60	0.72, 9.34	0.87	0.19, 4.05
Q4 (28.6–37.3)	19.80	4.46, 87.80	19.60	3.64, 105.51	7.42	1.90, 28.93	1.45	0.27, 7.67

Odds ratios (ORs) and 95% CIs relative to reference group (ref). ^a^Quarter (Q) 1 = lowest, Q4 = highest. ^b^Number of takeaway food outlets per 1000 local government area population. ^c^Proportion of all food retail outlets that are takeaway food outlets

Adjustment for deprivation attenuated many associations to non-significance. Local government areas with the greatest density of takeaway food outlets per 1000 population and the highest proportion, remained more likely to have a Specific Non-health planning policy. Those with the most takeaway outlets remained more likely to have a Specific Health planning policy (OR: 16.98; 95% CI: 1.44, 199.04).

#### Adult and children with excess weight

In the unadjusted model, compared to local government areas with the lowest proportion of 4–5 year olds with excess weight, those with the highest had 3.32 (95% CI: 1.30, 8.44) greater odds of Specific Health planning policy adoption (Table 4). Compared to local government areas with the lowest proportion of 10–11 year olds with excess weight, those with the highest had 2.96 (95% CI: 1.25, 7.02) greater odds of Specific Non-health, and 25.31 (95% CI: 6.74, 94.96) greater odds of Specific Health planning policy adoption. These associations were not significant in adjusted models.

**Table 4:** Associations of the proportion (%) of 4–5 and 10–11 year old children, and adults with excess weight in English local government areas and planning policy status, estimated using unadjusted and adjusted multinomial logistic regression models

	Specific Non-health Planning Policy Status				Specific Health Planning Policy Status			
	Unadjusted		Adjusted		Unadjusted		Adjusted	
	OR	95% CI	OR	95% CI	OR	95% CI	OR	95% CI
Excess weight								
Children 4–5 years (%) ^a, b^								
Q1 (0.0–20.2)	ref	–	ref	–	Ref	–	ref	–
Q2 (20.3–22.3)	0.94	0.45, 1.95	0.63	0.29, 1.39	0.64	0.22, 1.91	0.28	0.08, 0.98
Q3 (22.4–24.0)	1.28	0.57, 2.86	0.59	0.23, 1.47	2.04	0.75, 5.55	0.40	0.11, 1.37
Q4 (24.1–31.5)	1.05	0.46, 2.37	0.41	0.15, 1.08	3.32	1.30, 8.44	0.46	0.14, 1.55
Children 10–11 years (%) ^a, b^								
Q1 (0.0–29.3)	ref	–	ref	–	Ref	–	ref	–
Q2 (29.4–32.3)	1.52	0.71, 3.27	1.10	0.49, 2.48	2.50	0.58, 10.69	1.32	0.27, 6.28
Q3 (32.4–35.1)	2.31	1.07, 5.00	1.51	0.44, 2.95	8.33	2.20, 31.45	1.87	0.41, 8.58
Q4 (35.2–43.9)	2.96	1.25, 7.02	1.12	0.36, 3.51	25.31	6.74, 94.96	2.99	0.59, 14.91
Adults (%) ^a, c^								
Q1 (0.0–62.6)	ref	–	ref	–	Ref	–	ref	–
Q2 (62.7–66.0)	0.41	0.14, 1.17	0.44	0.15, 1.29	0.42	0.13, 1.35	0.43	0.11, 1.64
Q3 (66.1–68.3)	0.39	0.14, 1.12	0.36	0.12, 1.07	0.34	0.10, 1.15	0.35	0.09, 1.37
Q4 (68.4–76.2)	0.51	0.17, 1.48	0.33	0.10, 1.05	0.89	0.30, 2.64	0.48	0.13, 1.70

Odds ratios (ORs) and 95% CIs relative to reference group (ref). ^a^Quarter (Q) 1 = lowest, Q4 = highest. ^b^Child excess weight at 4–5 years and 10–11 years= BMI ≥85th percentile. ^c^ Adult excess weight = BMI ≥ 25 kg/m^2^

## Discussion

### Summary of findings

We conducted a cross-sectional analysis of the geographic distribution and clustering of English local government area planning policies for takeaway food outlet regulation, and associations between planning policy adoption and local characteristics. To our knowledge, this is the first time an analysis of this nature has been completed. Local government areas with adopted planning policies were geographically widespread. Those with health-focused planning policies often clustered together. Adoption of health-focused planning policies was associated with deprivation. After adjusting for deprivation, local government areas under Labour political control, that were predominantly urban, with highest numbers of takeaway food outlets, and those with more neighbouring and statistically similar areas also with health-focused planning policies in place, were most likely to have adopted health-focused planning policies.

### Interpretation of findings

Our spatial analyses identified a number of geographical clusters of local government areas with similar types of planning policy. This observation was supported by statistical analyses. These findings are suggestive of localised policy diffusion, underpinned by a tradition of knowledge sharing across local government in England. Such diffusion could also be exaggerated by formal working relationships between nearby local government areas. For example, through collaboration on sustainability and transformation plans or membership of Health and Wellbeing boards. The latter bring together professionals from neighbouring local governments to establish supra-local government area priorities for population health improvement [50–52]. Priorities agreed by one Health and Wellbeing Board could therefore relate to multiple local government areas and may result in observed planning policy clustering. Public health department structure may also play a role. Public health functions are sometimes shared by multiple local government areas with planning responsibility, which may perpetuate health-focused planning policy adoption across area boundaries. However, further research is required to better understand planning policy clusters, including how and why they form, and possible barriers to further planning policy diffusion.

Before adjustment for deprivation, a greater proportion of 10–11 year old children with excess weight was associated with health-focused planning policy adoption. Despite equivocal evidence regarding the relationship between takeaway food outlet exposure and childhood obesity in the research literature, this association could be due to a perception that children are vulnerable to food environment influences [53, 54]. There is also some evidence that children are more likely to purchase food in the vicinity of schools when food outlets are accessible [55]. This might help explain why the majority of health-focused planning policies aim to regulate takeaway food outlet proliferation on the school fringe [24]. Associations between the proportion of 10–11 year olds with excess weight and health-focused planning policy adoption were not observed after adjustment for deprivation. This may reflect that excess weight in children of this age is strongly associated with deprivation and should not be considered to negate the importance of this measure in considering planning policy adoption.

Areas with highest numbers of existing takeaway food outlets were more likely to have a health-focused planning policy. This observation may indicate a perceived need for regulation in response to high numbers of already established takeaway food outlets. However, planning policies in England can only regulate new takeaway food outlets, not existing ones. The current number already established of takeaway food outlets, despite being strongly linked with regulatory action, may therefore be an insufficient indicator of regulatory need. Stronger promotion of planning policy uptake in all areas, regardless of existing takeaway food outlet number, would serve as a more proactive use of planning to shape future healthy food retail access [56]. Predominantly urban and more deprived local government areas were also more likely to have adopted planning policies for takeaway food outlet regulation. These areas may appeal to prospective owners on the basis of perceived demand among more geographically concentrated and less affluent populations.

Conventional Labour political party ideology may broadly favour government-led regulation, whereas the Liberal Democrats may be more neutral, but not against government-led regulation [57]. This could help explain why areas under the political control of these parties were more likely to have health-focused planning policies. In contrast, areas under Conservative control, not traditionally aligned with government-led regulations [58], were no more likely to have a specific planning policy. Other explanations include that areas under Conservative control may be experiencing greater economic growth [59], resulting in a reluctance to adopt regulatory planning policies.

### Implications for policy, practice and future research

Adoption of planning policies for takeaway food outlet regulation, while in line with the National Planning Policy Framework [20], is currently at local discretion, and this appears to have resulted in incomplete policy uptake across England. Fragmented, locally implemented national guidance is not unique to the planning system in this context. For example, local government areas in England are responsible for tier-2 weight management services, and provision is similarly not widespread [60, 61]. If widespread use of planning to promote health is to be achieved, then reviewing and understanding the drivers of planning policy adoption could help inform practice. Overall, we found a number of local government area characteristics correlated with adoption of health-focused planning policies. The contribution of these characteristics to the decision to adopt a planning policy for takeaway food outlet regulation is currently unclear. Future work should explore the rationale behind planning policy adoption, and evidence considered in the adoption process. This understanding could be achieved by engaging with relevant planning and public health professionals.

### Methodological considerations

We completed an innovative analysis based on a national census of planning policy adoption across all local government areas in England. However, the study is not without limitations. The cross-sectional, observational design limits causal inference. Whilst we reviewed the most recently revised planning policy documents, we did not consider older documents where policies may have first been adopted and then carried forward to subsequent documents. Therefore, our outcome (planning policy adoption) may have preceded our exposure (local government area characteristic), but the extent to which this occurred is unknown. We were also unable to identify temporal trends in planning policy adoption or consider the evolution of planning policies since initial adoption.

Where possible, we included all local government areas in analyses. For takeaway food outlet and excess weight metrics, our sample was restricted due to differences between the date of this exposure data and year of planning policy document adoption. This said, our analytic samples of local government areas were broadly representative of all local government areas in England across key characteristics. Adult weight data were self-reported and could be prone to mis-reporting [62]. Finally, we did not adjust our statistical significance threshold to reflect the number of inferential tests carried out [63].

## Conclusions

In this analysis we characterised and mapped local government areas based on the adoption of planning policies for regulation of takeaway food outlets, identifying clusters of those with non-health and health focused planning policies. Geographical observations of clustering were supported by statistical analyses, with clusters possibly emerging due to the structure of local government. Political party in control, deprivation, urbanisation, and the actions of similar local government areas, were identified as characteristics correlated with planning policy adoption. Existing takeaway food outlet number and the proportion of children with excess weight were also associated with planning policy adoption and may serve as indicators of need. Future research should explore the deterministic role of these characteristics in conversation with local government stakeholders.

## Data Availability

The datasets used and/or analysed during the current study are available from the corresponding author on request.
